# Renewable Biopolymers Combined with Ionic Liquids for the Next Generation of Supercapacitor Materials

**DOI:** 10.3390/ijms24097866

**Published:** 2023-04-26

**Authors:** Julia L. Shamshina, Paula Berton

**Affiliations:** 1 Fiber and Biopolymer Research Institute, Department of Plant and Soil Science, Texas Tech University, Lubbock, TX 79409, USA; 2 Chemical and Petroleum Engineering Department, Schulich School of Engineering, University of Calgary, Calgary, AB T2N 1N4, Canada

**Keywords:** biopolymers, EDLCs, flexible electrodes, gel polymer electrolytes, ionic liquid electrolytes

## Abstract

The search for biocompatible and renewable materials for the next generation of energy devices has led to increasing interest in using biopolymers as a matrix component for the development of electric double-layer capacitors (EDLCs). However, using biopolymers as host matrices presents limitations in performance and scalability. At the same time, ionic liquids (ILs) have shown exceptional properties as non-aqueous electrolytes. This review intends to highlight the progress in integrating ILs and biopolymers for EDLC. While ILs have been used as solvents to process biopolymers and electrolyte materials, biopolymers have been utilized to provide novel chemistries of electrolyte materials via one of the following scenarios: (1) acting as host polymeric matrices for IL-support, (2) performing as polymeric fillers, and (3) serving as backbone polymer substrates for synthetic polymer grafting. Each of these scenarios is discussed in detail and supported with several examples. The use of biopolymers as electrode materials is another topic covered in this review, where biopolymers are used as a source of carbon or as a flexible support for conductive materials. This review also highlights current challenges in materials development, including improvements in robustness and conductivity, and proper dispersion and compatibility of biopolymeric and synthetic polymeric matrices for proper interface bonding.

## 1. Historic Perspective of IL Electrolytes for Electric Double Layer Capacitors (EDLCs)

### 1.1. Performance Requirements for EDLCs: The Emergence of ILs as Electrolytes 

Electrochemical capacitors (also called supercapacitors) are an important segment of the clean energy portfolio due to their high-power density and long cycle life. The energy storage mechanism in supercapacitors is based on the electric charge buildup by a charge–discharge process (electrosorption) at the electrode–electrolyte interface and/or redox reactions on the electroactive surface. The energy storage in supercapacitors occurs via two complementary types of contributions, i.e., contributions from electric-double layer capacitance and contributions from pseudocapacitive faradaic processes. The distribution of these two types of capacitances depends on both the material and structure of the electrodes. Hybrid pseudocapacitors utilizing a faradaic process at one electrode and a purely capacitive component at the other are also known. In addition to the electrode material, the electrolyte is an important part of the supercapacitor and affects both the energy and power performance of the device. 

Efforts to miniaturize supercapacitors have mainly focused on purely electric double-layer capacitors (EDLCs)—high power density devices, using electrodes with high surface areas based on traditional porous carbon, graphene [1], reduced graphene oxide/carbon nanotubes [2], onion-like carbon [3], etc. A few miniaturized systems have used RuO_2_ [4] or conjugated polymers [5,6,7] to add a pseudocapacitive component to the energy-storage system, although these polymers are not optimal for high-energy density systems.

Miniaturized supercapacitors present two fundamental challenges: 1. While the power density is high, the energy density is low, and 2. The current is delivered at a continuously decreasing voltage. The power (P) of EDLCs is proportional to the operating voltage (V) and inversely proportional to the internal resistance (ESR). While ESR depends on the design of the ultracapacitor and the types of electrodes, V depends on the stability of the electrolyte at the applied potential. Thus, a proper choice of electrolytes is the most effective way to increase both the energy and the power of EDLCs. However, studies have shown that it is not possible to fabricate EDLCs with voltages higher than 3 V using conventional organic electrolytes. In addition, conventional electrolytes suffer from operational safety problems related to volatility and flammability. Hence, in the last decade, much effort has been dedicated to the development of safer electrolytes with larger electrochemical windows (>3 V).

Electrolytes based on ionic liquids (ILs) are the most promising [8]. In fact, the emergence of the IL field is, in part, a legacy of the United States Air Force efforts that conducted electrochemical studies using room-temperature molten salts [9,10]. ILs offer unique material properties, including thermal and chemical stability, a broad electrochemical window, high ionic conductivity, and non-flammability. All these properties can be easily modified by tuning the component ions of IL electrolytes [11,12,13,14]. Redox-stable ILs with large electrochemical windows have led to a resurgence in interest in electrochemical applications with promising technological applications [15,16,17]. 

As electrolytes, ILs have preferential characteristics such as high conductivity (mostly up to 2 S m^−1^, with exceptionally high conductivities of 10 S m^−1^ being reported for imidazolium-based ILs [18]). A comprehensive analysis and correlation of IL structure vs. conductivity data for energy applications indicated that most of the evaluated ILs presented conductivities within the range of 7 × 10^−3^ to 7 S m^−1^, whereas a conductivity of the ‘classic’ battery electrolyte Li[PF_6_] solution ranges from 0.7 to 1.5 S m^−1^ [19]. The second attractive property of the ILs is their wide electrochemical stability window (up to 6.3 V [20,21,22,23,24,25]), with no degradation of electrochemical performance compared with the traditional electrolytes. Another important bulk characteristic of ILs is their static dielectric constant (ε_S_), a characteristic that describes ILs’ solvation capability, which depends on the cation–anion combination and has been determined in aprotic ILs with [NTf_2_]-anion to be in the range of 12–15.8, and in ILs with [C_2_mim]-cation 11.7–35.0 [20]. Static dielectric constants of some protic ILs have ε_S_ values up to 85.6 [20]. On the other hand, the (often) high viscosity of the ILs might hinder charge transport and lower transference number. In addition, the capacitance/behavior of ILs at electrodes (types of electrodes and the layering behavior of the IL electrolyte) must be taken into consideration for supercapacitor applications. 

Additional properties that make ILs attractive for electrolyte applications include high thermal stability, low vapor pressure, and tailored properties due to the large number of cation/anion combinations. The effects of cations, anions, the length of the alkyl chain, etc. have been extensively studied. However, most applied research still focuses on the ‘common’ cations and anions in IL electrolyte (Table 1): [C_2_mim]^+^, [C_4_mim]^+^, [C_1_C_4_Pyr], [N_2222_]^+^ paired with bis(trifluoromethyl)sulfonamide ([NTf_2_]^−^), dicyandiamide ([DCA]^−^), trifluoromethanesulphonamide ([OTf]^−^), hexafluorophosphate ([PF_6_]^−^), and tetrafluoroborate ([BF_4_]^−^). Furthermore, many ILs are now commercially available in ‘electrochemical purity’ and can be straightforwardly prepared on large scales, making their consideration in this type of application feasible. Ionic liquids electrolytes are currently under either pilot- or commercial-scale production by numerous companies, including C-Tech Innovation (IL electrolytes for aluminum electroplating, pilot scale), G24 Power and H. Glass (IL electrolytes in dye-sensitized solar cell, commercial and pilot scale, respectively), IoLiTec (IL electrolytes for electrochromic windows, pilot scale, aluminum electroplating, pilot scale, and sensors, commercial scale), NantEnergy, NOHMs Technologies, and Pionics (IL electrolytes for zinc-air and lithium-ion battery, respectively, commercial and pilot scale), Novasina (IL electrolytes in gas sensor, commercial scale), and Panasonic (supercapacitor applications, commercial scale) [21].

**Figure 1:**
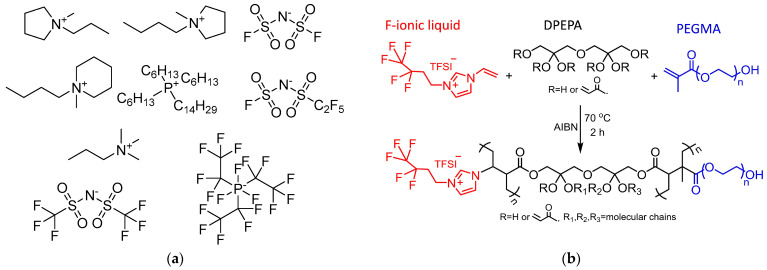

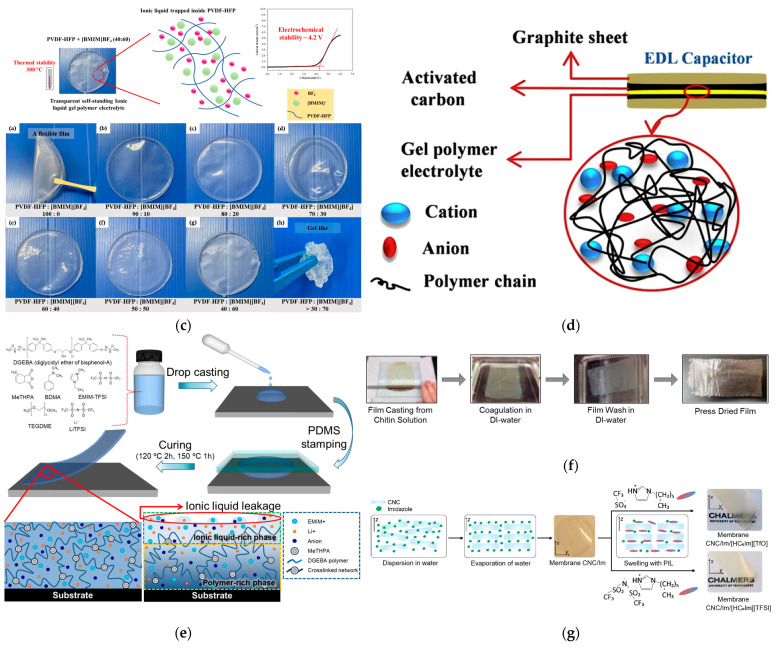
(**a**) Ions used for IL electrolytes; (**b**) Synthesis of GPE containing ILs as end groups (monomers identified with different colors). Reproduced with permission from [31]; (**c**) GPE formed from poly(vinylidene fluoride–hexafluoropropylene) (PVDF–HFP) with entrapped 1-butyl-3-methylimidazolium tetrafluoroborate ([C_4_mim][BF_4_]). Reproduced from [32] (Open access CC-BY license); (**d**) Generalized construction of solid-state gel polymer electrolytes based on ILs containing imidazolium cations and tetrafluoroborate anions for electrochemical double-layer capacitors. Reproduced with permission from [33]; (**e**) Fabrication of a flexible GPE film. The mechanical support matrix for [C_2_mim][TFSI]/Li[TFSI] was created by casting a diglycidyl ether of bisphenol-A (DGEBA) epoxy resin, followed by curing with a methyl tetrahydropthalic anhydride (MeTHPA) with *N*-benzyldimethyl-amine (BDMA) catalyst, followed by plasticization. Reprinted with permission from [34]; (**f**) Chitin film casting; (**g**) Schematic procedure to obtain flexible CNC membranes with polymeric ILs and IL electrolytes. Reproduced from [35] (Open access CC-BY license).

Initial studies of IL electrolytes showed that electrochemically stable IL cations, such as imidazolium, ammonium, pyrrolidinium, pyridinium, or phosphonium, could be paired with [NTf_2_]^−^ or other fluorinated anions, resulting in ILs with low melting points and high air- and water-stability, with electrochemical windows larger than 5 V. It was also shown at that time that the stability of the IL towards reduction was limited by the cation, not the anion. Early representative examples of the electrochemical properties of “pristine ILs” as electrolytes are shown in Table 1 and Figure 1a.

### 1.2. Next Generation of Electrolytes: Gel Polymer Electrolytes (GPEs) and Solid Polymer Electrolytes (SPEs)

The “pristine IL” electrolytes are suitable for supercapacitors applications, but they are difficult to confine. This limits their applications in flexible and printed electronics. Hence, after this initial trend of using “pristine ILs” as electrolytes and determining which ILs work best (see Table 1) from an electrochemical standpoint, gel polymer electrolytes (GPEs, also called quasi-solid-state electrolytes) and solid polymer electrolytes (SPEs) were used to incorporate the “pristine ILs” into their structures. SPEs, defined as the solvent-free salt solution in a polymer host material, are of great interest due to their wider range of applications, such as fuel cells, solar cells, batteries, sensors, and electrochemical capacitors. They offer numerous advantages, for example, wider electrochemical and thermal stability range, as well as low volatility and easy handling [12]. However, they exhibit relatively low ionic conductivity compared to liquid electrolytes. Therefore, the current research is mainly focused on GPEs, which exhibit liquid-like ionic conductivity. GPEs consist of a conductive liquid phase embedded within a polymer matrix and are divided into four areas: aqueous gel electrolytes, non-aqueous gel electrolytes, IL-based gel electrolytes, and redox gel electrolytes [36,37,38,39]. The following sections will focus on biopolymeric IL-based GPE systems. 

GPEs networks are made of polymeric ILs (PILs) (ionogels, Figure 1b) or synthetic polymers (Figure 1c) [40,41,42,43] as hosts for the entrapment of the ILs [41]. Namely, GPE consists of ILs *within* a polymer matrix, and hence exhibits both solid-like characteristics and liquid-like diffusivity, coupled with a large operation window. Examples of polymers used in this type of application include polyethylene oxide (PEO), poly(methylmethacrylate)-co-poly(ether glycol) (PMMA-co-PEG), polyethylene glycol (PEG), and poly(vinylidenefluoride-co-hexa-fluoropropylene) (PVdF-co-HFP)), whereas poly(IL networks) include poly(diallyldimethylammonium) bis(trifluoromethanesulfonyl)imide ([PDADMA][NTf_2_]) [41], poly(1-[2-(2-(2-(Methacryloyloxy)ethoxy)ethoxy)ethyl]-3-methylimidazolium bis(trifluoromethylsulfonyl)imide)-co-poly(poly(ethylene glycol) methyl ether methacrylate) [42], etc. While these types of electrolytes possess wide electrochemical windows [44], exhibit high conductivity, are highly compatible with electrodes, and present improved safety features compared to “pristine” ILs, they have notable limitations of low(er) mechanical strength because they still require the use of “pristine ILs” for elevating the ionic conductivity.

In parallel, SPEs incorporating ILs have been developed and reviewed [45,46] (e.g., Figure 1d,e), where poly(vinylidene fluoride-co-hexafluoropropylene) (PVdF-co-HFP) was the most commonly-used polymer matrix. The following systems are known: PMMA/1-butyl-3-methylimidazolium bis(trifluoromethanesulfonyl)imide ([C_4_mim][NTf_2_]) [47], polyacrylonitrile (PAN)/[C_4_mim][NTf_2_] [47], poly(vinyl alcohol) (PVOH)/1-butyl-3-methylimidazolium chloride ([C_4_mim]Cl) [48], PVdF-co-HFP/1-methyl-3-propylimidazolium bis(trifluoromethanesulfonyl)imide ([C_3_mim][NTf_2_]) [49], PVdF-co-HFP/1-methyl-3-butylimidazolium tetrafluoroborate ([C_4_mim][BF_4_]) [50], PVOH/1-methyl-3-butylimidazolium bromide ([C_4_mim]Br) [51], PVdF-co-HFP/1-methyl-3-butylimidazolium dicyanamide ([C_4_mim][DCA]) [52], PVOH/([C_4_mim][NTf_2_], PVdF-co-HFP/1-propyl-3-methylimidazolium tetrafluoroborate ([C_3_mim][BF_4_]) [52], PVdF-co-HFP/1-methyl-3-propylimidazolium triflate ([C_3_mim][OTf]) [53], and PVdF-co-HFP/1-butyl-3-methylimidazolium triflate ([C_4_mim][OTf]); some of these systems used salt additives (Mg(OTf)_2_, [NH_4_][CH_3_COO], etc.). However, the performance was restricted due to the poor conductivity at room temperature of the SPEs [54,55]. In addition, these polymer matrices were mainly composed of nondegradable synthetic plastics. 

## 2. Can Biopolymers Be Used in the Design and Development of IL Electrolytes?

Recently, an increasing trend in the application of biopolymer materials as matrices in the gel- or solid-electrolytes is seen in the literature. Biopolymers, unlike synthetic polymers, feature renewability, eco-friendly origin, and cost-effectiveness, while being mechanically, chemically, and thermally stable. Examples include biomass-based electrolytes from cellulose or its derivatives [56], chitin/chitosan [57,58], starch [59], alginate, and so on. Biopolymer-based constituents can be surface-modified and appended with various functionalities to produce different kinds of materials [11]. Cellulose and chitin, the two most abundant biopolymers in nature, are extensively used as polymeric matrices. The reason that most matrices for electrolyte support in EDLCs are still of synthetic origin is that biopolymeric alternatives have not been commercially available, manufacturing of some is limited by significantly higher prices compared to synthetic plastics, difficulties in making composite matrices, and unmet performance expectations. Ionic liquids have been proposed to overcome some of those limitations.

In general, IL-based solid electrolytes are commonly known as ionogels. Ionogels are synthesized by the incorporation/entrapment of ILs in any inorganic- or/and organic-type solid host materials. The use of ILs has been proposed in the following areas: 1. As solvents to process biopolymers [60], and 2. As electrolytes [61]. In the former case, gel membranes are prepared by dissolution, crosslinking, and casting the solution of biopolymer dissolved in the IL (Figure 1f). After the membrane preparation, membranes are usually immersed into the IL electrolyte to form GPEs which are highly conductive and demonstrate superior ion transference and low interfacial resistance. The mechanical and electrochemical properties of the gel membranes can be easily tuned by the extent of crosslinking. 

ILs are used as electrolytes in solid supports either by direct polymerization of ILs to form PILs, or post-modification of (bio)polymeric hosts with (electrolyte) ILs. Using polymerized ILs could, on the other hand, compromise the ionic conductivity of the material in comparison to that of monomeric ILs. Therefore, there has been increasing demand for noncovalent immobilization of ILs on the solid support without compromising their unique properties. The biopolymers and (electrolyte) ILs are usually mixed in the presence of an organic solvent, and the SPEs/GPEs are formed by casting, followed by solvent evaporation. Depending on the IL selected, the IL might weaken the interactions between the biopolymeric chains and the electrolytes, reducing the solvation of the cations, and eventually promoting the decoupling of ions. In addition, the IL acts as a plasticizer, “softening” the polymeric architecture and enhancing the flexibility of the polymer system, accelerating the polymer segmental mobility and ionic transportation by providing more conducting pathways. The inclusion of IL also destroys the ordered arrangement of the biopolymer backbone, and thus decreases the crystallinity of the polymer matrix. In other words, it increases the amorphousness of the polymer electrolytes and leads to high ionic conductivity by providing more voids and free spaces for ionic migration. On the other hand, increasing the amorphous fraction might compromise its mechanical properties. 

Although including the electrolyte ILs in the biopolymeric matrix results in an increase in the ionic conductivity, the conductivity reaches a maximum due to the agglomeration of mobile ions with further addition of ILs, and, hence, leads to the formation of ion pairs, which inhibit the mobility of free charge carriers and thus prevent the passage of ions in the polymer matrix. As a result, the initial IL-biopolymer electrolytes reported showed limited ionic conductivity (Table 2). Typically, the ionic conductivity of an electrolyte for a supercapacitor can range from as low as 1 mS cm^−1^ to as high as 100 mS cm^−1^ or more. Values above 10 mS cm^−1^ are desirable for most supercapacitor applications. The selection of the electrolyte IL will define the operative electrochemical window for the supercapacitor. For example, a triflate-based biopolymer electrolyte depicted a wider electrochemical window compared to that of the hexafluorophosphate system (from −1.4 to 1.5 V for [C_4_mim][PF_6_] vs. −1.5 to 1.6 V for [C_4_mim][OTf]) [62], while electrochemical windows of 3.0 and 3.9 V were reached using [C_2_mim][BF_4_] [63] and [C_2_mim][SCN], respectively [64].

A quick overview of Table 2 highlights the use of ILs able to interact with the biopolymers through hydrogen bonding, resulting in their encapsulation in the biopolymeric matrices. For example, the IL *N*-butyl-*N*-methylpyrrolidinium bis(trifluoromethylsulfonyl)imide ([Pyrr_1,4_][NTf_2_]) was entrapped into a methyl cellulose (MC) matrix after co-dissolution in, and subsequent removal of, *N,N*-dimethylformamide (DMF) [74]. Room temperature moduli >1 GPa were achieved for the resulting gels, for all compositions with <60% [Pyrr_1,4_][NTf_2_]. At [Pyrr_1,4_][NTf_2_]/MC = 90/10, conductivities of 1.4 mS cm^−1^ at 30 °C, 6 mS cm^−1^ at 90 °C, and 11.3 mS cm^−1^ at 140 °C, a factor of ~3 less than the neat [Pyrr_1,4_][NTf_2_], were achieved with films that had moduli of 150, 109, and 57 Mpa, respectively.

As can also be seen in Table 2, only a few materials reached the high ionic conductivity required for superconductor materials. For example, the use of chitosan incorporated with [C_2_mim][BF_4_] IL in EDLCs with activated carbon electrodes has shown enhanced discharge capacitance and better rate performance compared to that using only liquid [C_2_mim][BF_4_] [76]. This was attributed to the high affinity of chitosan for the activated carbon electrode and the higher ionic conductivity of the gel electrolyte. The cell did not show any sign of degradation even after 5000 cycles. This same IL was used for embedding into cellulose and chitin films cast from a [C_2_mim][OAc] solution [63], resulting in an electrolyte with ionic conductivities of 21 mS cm^−1^ which, when used in a supercapacitor setup, resulted in a stable cell with capacitance retention of 90% after 10,000 cycles. 

Cellulose was also dissolved in the IL [C_4_mim]Cl, mixed with a solution of chitin in 1-allyl-3-methylimidazolim bromide ([AMIm]Br), and cast onto a glass plate to produce a film. The film was then immersed in an H_2_SO_4_ solution [78]. The acidic hybrid gel with ILs–H_2_SO_4_ showed high ionic conductivity, comparable to that of the aqueous H_2_SO_4_ electrolyte (57.8 vs. 61.9 S m^−1^ at 25 °C, respectively). When the GPE was used in an ELDC cell with activated carbon fiber cloths, the discharge capacitances in the hybrid gel with ILs–H_2_SO_4_ and the aqueous H_2_SO_4_ solution were 162 and 155 F g^−1^, respectively. The hybrid gel with ILs–H_2_SO_4_ retained 80% of its initial value even after 100,000 cycles. At an elevated temperature of 60 °C, the discharge capacitance for an EDLC cell with the acidic gel at 100 mA g^−1^ was estimated to be 300.0 F g^−1^, which is 27.1% higher than the corresponding value at room temperature (25 °C) [82]. Even at a low temperature of 5 °C, the respective ionic conductivities for the gel and the aqueous H_2_SO_4_ solution were 43.3 and 46.0 S m^−1^. 

The preparation of ionogels incorporating both ILs and biopolymers allowed generation of flexible, mechanically stable materials that could be used as biopolymeric electrolytes. For example, [C_4_mim]Cl was mixed with chitosan powder and hydroxyethyl methacrylate (HEMA) [77]. Light-induced polymerization was used under UV irradiation to obtain the [C_4_mim]Cl/chitosan/pHEMA GPE. The resulting GPEs showed values of Young’s modulus up to 34 kPa. Below the melting point of [C_4_mim]Cl (76 °C), the conductivity values of the gel slowly increased with temperature, whereas from 80 to 200 °C, sharp increases in ionic conductivities were observed (from 31 to 81 mS cm^−1^). A supercapacitor was then built using commercial activated charcoal as electrode material. The ionogel-based supercapacitor showed a high capacitance of 165 F g^−1^ tested at 200 °C with a current density of 1.5 A g^−1^, much higher than the 15 F g^−1^ at a current density of 0.1 A g^−1^ measured at room temperature. The specific capacitance also increased from 4 to 104 F g^−1^, following the enhanced conductive trends at various temperatures. Encouragingly, the supercapacitor showed excellent capacitance retention over 2000 charge–discharge cycles at 100 °C with a constant current density of 0.5 A g^−1^.

Nanocrystalline materials can also be used instead of microcrystalline biopolymers. Mixing conductive IL electrolytes into the nanocrystalline substrates allows to produce conductive composites (Figure 1g). In this case, a self-encapsulation effect of the ILs is observed, which improves not only high IL electrolyte loading, but also strength, due to the high aspect ratio and outstanding mechanical properties of nanocrystals. In addition, nanocrystals can be effectively dispersed in the polymeric matrix and facilitate ion transport by increasing ion mobility and salt dissociation. For example, shape-persistent conductive GPEs were prepared from sulfonated cellulose nanocrystals (CNCs)/hyperbranched PILs (with an asymmetrical peripheral composition consisting of hydrophobic n-octadecylurethane arms and hydrophilic, ionically-linked poly(N-isopropylacrylamide), [PNIPAM]) as a supporting matrix, with incorporated 1-ethyl-3-methylimidazolium bis(trifluoromethanesulfonyl)imide ([C_2_mim][NTf_2_]) IL [79]. The structure of the nanocrystal/PIL matrix was capable of confining 95 wt% IL without losing its self-sustained shape. The resulting ionogels possess high mechanical strength and elastic modulus (≈5.6 MPa) while preserving the ionic conductivity of the IL (≈7.8 mS cm^−1^ for the biopolymeric materials vs. ≈ 10.9 mS cm^−1^ for the IL [C_2_mim][NTf_2_]). This same design, i.e., using the same PIL and electrolyte IL, was used, but combining both cellulose nanofibers (CNFs) and CNCs, and the resulting matrices were able to hold 90 wt% of the electrolyte IL [81]. Despite the high ionic liquid content (88–90 wt%), the composite ionogels exhibited a high Young’s modulus (>0.9 GPa) and tensile strength (>11.0 MPa). Ionic conductivities were also superior, in the range of 4.3–6.2 mS cm^−1^. When used in a supercapacitor setup, capacitance values of 34.5–44.1 F g^−1^ at 100 mV s^−1^ were observed, with up to 85% capacitance retention upon 10,000 cycles.

In addition, CNCs can be grafted with polymers for further loading with IL electrolytes instead of dispersion. The poly(2-phenylethylmethacrylate) (PPMA)-grafted nanocellulose nanocomposite film was then loaded with 30 wt% 1-hexyl-3-methylimidazolium bis(trifluoromethanesulfonyl)imide ([C_6_mim][NTf_2_]) [81]. These films with [C_6_mim][NTf_2_] not only displayed remarkable improvements in toughness (>25 times) and tensile strength (>70 times) relative to the corresponding films consisting of the ionic liquid embedded in the two-component CNC/PPMA nanocomposite, but also showed higher ionic conductivity than the corresponding neat PPMA with the same weight percentage of IL (5.7 ± 2.1 × 10^−3^ vs. 3.4 ± 1.8 × 10^−3^ mS cm^−1^ at 30 °C, respectively). Notably, the ionic conductivity of the CNC/PPMA–[C_6_mim][NTf_2_] film decreased at ~60 °C as a consequence of the lower critical solution temperature phase transition of the grafted polymer in the IL, which led to phase separation. However, holding the film at room temperature for 24 h returned the film to its original homogenous state. These materials exhibit properties relevant to thermal cutoff safety devices (e.g., thermal fuse) where a reduction in conductivity above a critical temperature is needed.

## 3. Biopolymeric Carbon as Electrode and ILs as Electrolytes

In addition to using biopolymers as main components in solid electrolytes, biopolymers can be used to create electrodes that would be advantageous as a freestanding and binder-free type of electrode for flexible supercapacitors. While this review covers advances that combine IL electrolytes and biopolymers within a single device, interested readers are referred to comprehensive reviews that focus on the development of biopolymeric materials (e.g., nanocellulose, chitin-derived nitrogen-doped porous carbons with ultrahigh specific surface area, chitosan porous carbon composites) in supercapacitor applications [83,84,85].

Because a conventional binder uses synthetic polymers [86,87], it can be replaced with a biopolymer-based binder consisting of cellulose-based materials [88], chitin/chitosan [89,90], lignin [91], etc., able to hold other materials together and exhibiting good bonding properties with the current collector. ILs can *simultaneously* dissolve biopolymers and stabilize nanoparticles. For instance, graphene is rich in π-electrons, and it has been shown that strong IL cation–π interactions exist between carbon nanomaterial and IL with aromatic cations such as imidazolium. It was recently shown with computational studies that the interaction of the ILs with the graphitic surfaces is considerably influenced by the charge transfer between the component ions [92]. Multiple approaches that allow the formation of composites of various biopolymers with nanomaterials to produce films and membranes have been reported, utilizing activated carbon [93], graphene [89,94], graphene oxide [95], polypyrrole (PPy) [96], multiwalled carbon nanotubes (MWCNTs) [97], etc. Still, only a few reports can be found using biopolymer-based electrodes in combination with ILs as electrolytes. For example, cellulose was dissolved in the IL [C_4_mim]Cl and combined with PPy and trihexyl(tetradecyl)phosphonium bis[(trifluoromethyl)sulfonyl] amide ([P_66614_][NTf_2_]) as the IL plasticizer to form PPy–Cellulose–IL composite films via light-induced polymerization [96]. 

Porous carbon is one of the best electrode materials to date and found to be widely used to synthesize an efficient supercapacitor device. Porous carbon has outstanding properties such as higher surface area, larger pore volume, great electrical conductivity, and efficient chemical stability. Also, it is environmentally friendly and can be made from any organic matter on Earth. A simple, costless, and nonpolluting strategy was proposed by using the decomposable and water-removable NaNO_3_ salt crystals as both the template and pore engineer in the gelatin biopolymer aerogel to fabricate a cross-coupled macro-mesoporous carbon material, with high surface area (approaching 3000 m^2^ g^−1^) [98]. When combined with [C_2_mim][BF_4_] as the electrolyte, a high energy density of 92 Wh kg^−1^ was obtained at 1 kW kg^−1^, and remained at 39 Wh kg^−1^ even when the power density increased up to 200 kW kg^−1^, outperforming nearly all hitherto reported porous carbon at high current density. The calculated specific capacitances of the material were 166, 152, 142, 135, 127, 118, 111, 98, 84, and 70 F g^−1^ under the current densities of 0.5, 1, 2, 5, 10, 20, 30, 50, 75, and 100 A g^−1^, respectively. Alternatively, porous carbon was prepared from cornstarch biopolymer by a simple carbonization process, initially at 800 °C [99]. The resulting material was coated with a polymer electrolyte film of PVDF–HFP, doped with 300 wt% of 1-ethyl-3-methylimidazolium tricyanomethanide ([C_2_mim][TCM]) IL as a separator. A supercapacitor device was then fabricated at a laboratory scale with the prepared porous carbon electrodes sandwiched around the electrolyte film, which yielded a specific capacitance of 188.4 F gm^−1^ at 10 mHz, confirmed from the electrochemical low-frequency impedance spectroscopy plot. Cyclic voltammetry results showed a high specific capacitance of 184.8 F gm^−1^ at 5 mV s^−1^.

Another approach is the thermal processing of different lignin grades into high-performance carbon materials [100,101,102]. Activated porous lignin-based carbons with specific high surface areas of more than 1800 m^2^ g^−1^ were recently synthesized by employing a simple two-step process, which consisted of a high-temperature thermal treatment of a lignin/KOH composite under an inert gas atmosphere without any addition of templating agents; the synthesis was followed by a washing step to remove byproducts of the activation procedure [103]. The electrochemical performance of the resulting carbons indicated that these could be used as an active material in double-layer capacitors, using the IL [C_2_mim][BF_4_] as the electrolyte to enhance storage ability. A capacitance of 231 F g^−1^ at 1 A g^−1^ and 203 F g^−1^ when the current was increased 10-fold to 10 A g^−1^ was achieved for carbon with a specific surface area of more than 1800 m^2^ g^−1^. One of the most crucial factors determining the electrochemical response of the active materials was found to be the strong surface functionalization by oxygen-containing groups. However, over the course of 10,000 charging−discharging cycles, a decay in capacitance of about 50% was observed, which might be due to the large voltage window and the surface functionalization.

A novel material platform based on choline IL-functionalized biopolymers, which can form a hydrogel electrolyte when exposed to visible light, was also developed. The polymer electrolyte entailed mixing a methacrylate polymer (gelatin methacryloyl, GelMA, or polyethylene glycol diacrylate, PEGDA) and choline acrylate ([Cho][Acrylate]) to make [Cho][Acrylate]–GelMA (BG) and [Cho][Acrylate]–PEGDA (BP) hydrogels, respectively [104]. Lithium phenyl-2,4,6-trimethylbenzoylphosphinate (LAP) was added as a photoinitiator, and visible light 405 nm was used for 60 s. Graphene hydrogel was prepared by reducing graphene oxide with ascorbic acid for use as electrodes. Fine-structure, interdigitated, biocompatible, and implantable soft micro-supercapacitors (MSC) were created by 3D in situ bioprinting of these polymer electrolytes in combination with rheologically-optimized graphene hydrogel–laponite (GH–L) blend as electrode material. The hydrogel electrolyte had a specific capacitance of ~200 F g^−1^, while the MSC had a specific capacitance of ~16 μF g^−1^ at a current density of 1 A g^−1^, volumetric capacitance of ~44 μF cm^−3^, cyclic stability up to 10,000 cycles, energy densities nearly as high as implantable batteries, and the power density level of implantable supercapacitors. This novel material platform enables in situ 3D printing of flexible bioelectronics structures with an integrated life-long power source.

In all aforementioned cases, it is important to note the electrostatic adsorption of ionic species at the interface of electrode and solution in EDLCs. Electrolytes are typically confined in the pores of electrodes (e.g., carbon), where the confinement region is often smaller than 1 nm, so that resulting pore sizes are only one or two times the diameters of bare ions or solvent molecules [105]. (The ionic size of the common cation and anion of IL electrolytes—[C_2_mim]^+^, [C_4_mim]^+^, [C_1_C_4_Pyr], [N_2222_]^+^ paired with fluorinated [NTf_2_]^−^, [DCA]^−^, [OTf]^−^, and [PF_6_]^−^—can be found in reference [39].) Hence, the in-pore composition of ions is anticipated to differ from the bulk IL: the ions closest to the electrodes can adsorb and order at the electrode surface [106]. Properties of the system (e.g., charging rates) could be affected by pore width due to variations in initial ion populations, and could be adjusted by modification of the pore diameter of the electrodes. In electrodes with a sub-nanometer size, the pores (so-called ionophobic pores) that contain a small number of ions at zero charge bring about enhanced power and energy density in supercapacitors [107], due to an increase in ion solvation. Recent molecular dynamics simulations on two IL electrolytes, [C_2_mim][BF_4_] and [C_4_mim][NTf_2_] (1.5 M in acetonitrile), demonstrated that, under confinement between graphene sheets forming slit pores of various widths, in-pore mole fraction of the IL varies from nearly 0 (pore width 0.76 nm) to 0.3 (pore width 0.84 nm), then again decreases to almost 0 (pore width ~1 nm), and then rises again, to accommodate average IL mole fraction of 0.12. Interestingly, the IL composition was important: for [C_2_mim][BF_4_]/acetonitrile solution (IL cation width 0.76 nm, anion size 0.45 nm [39]), ions were nearly fully excluded from pores with widths near 1 nm, while for [C_4_mim][NTf_2_] (IL cation width 0.90 nm, anion size 0.79 nm [39]), only a slight depletion of ions was observed. In addition, the IL size asymmetry should be considered: the spontaneous structure formation at the interface of electrode/electrolyte is affected by ionic size asymmetry, which plays a significant role in charge screening and, hence, ionic density [108].

## 4. Discussion & Conclusions

IL-type electrolytes have shown to be the most promising, safe(r) materials to achieve EDLCs with voltages higher than 3 V, due to their high thermal (>200 °C, often 300 °C), chemical and electrochemical (>5 V) stability, and high ionic conductivity (up to 10^−3^ S cm^−1^). However, the applications of neat ILs in flexible and printed electronics are limited due to potential leakage problems. As a result, (electrolyte) ILs are often integrated with synthetic polymers to form SPEs or GPEs. An obstacle to SPE usage as electrolytes in EDCLs is their relatively low ionic conductivities at room temperature, whereas IL-based GPEs are not only compatible with IL electrolytes but exhibit ionic conductivity equivalent to that of the liquid type. GPEs also offer long-term stability and superior performance. However, GPEs have somewhat compromised mechanical strength due to (usually) a large amount of entrapped IL electrolyte. To compensate for the loss of mechanical properties due to the addition of ILs, either PILs are used, or inert, often nanosized, fillers are added to GPEs.

At the same time, diverse technologies for energy storage are currently utilizing renewable materials (biobased and recyclable) that can provide novel chemistries and meet different end-application demands, both in supercapacitor and battery applications. Biopolymers are currently used as 1. (electrolyte)-IL supports, playing the role of host polymeric (or co-polymeric) matrices, 2. polymeric fillers, often in nanosized form, and 3. backbone polymer substrates for further grafting of synthetic polymers via ‘grafting to’ methods, forming IL-embedding matrices [81]. Among these, supported IL-biopolymer electrolytes show limited ionic conductivity due to the ion agglomeration at high IL load and formation of ion pairs which restrict the mobility of free charge carriers. Contrarily, the use of biopolymers as free fillers results in the synergistic effect of the polymer–filler system, and often leads not only to superior mechanical stability, but also improved performance. The reason for this is that the filler improves the free volume via the expansion of polymer chains in the GPE, resulting in the creation of micro-channels for ion mobility.

As interconnected reinforcing nanofiber networks (bacterial cellulose and CNFs) are frequently utilized to construct robust GPEs with high mechanical strength (i.e., stretchable), durability, anti-freezing properties, and thermal stability over synthetic polymers. In addition, the incorporation of such networks improves the quality of the interface between the electrolyte and the electrode. Finally, polymer chains grafted on the CNCs/CNFs enhance the mechanical properties of the composite matrices due to chain entanglements, producing polymer-grafted celluloses, that, upon (electrolyte)-IL loading, become mechanically robust and highly conductive. For example, PMMA-grafted CNCs (via grafting of PPMA to carboxylic acid-functionalized CNCs), followed by embedding [C_6_mim][NTf_2_], resulted in a formation of relatively strong ion-conducting films [81]. The films displayed remarkable improvements in tensile strength (>70 times) and enhanced toughness, compared to the corresponding films consisting of the IL embedded in the two-component CNC/PPMA, prepared by simple mixing.

In addition to utilizing biopolymers as main components in GPE/SPE electrolytes, biopolymers can be used to create freestanding and binder-free types of electrodes with incorporated carbon, graphene/graphene oxide, etc. These types of flexible electrodes provide high capacitance, high-rate capability, low self-discharge, and cycling stability.

Even with all these advantages, reports on GPEs with incorporated biopolymers are still limited, with no biopolymer–IL systems for such applications yet commercialized. Currently, studies are still focused on the fundamental network construction to achieve the improvement in strength, stretchability, freezing resistance, etc. and the optimization of physicochemical properties of biopolymer-containing electrolytes for electrochemical energy devices.

Challenges include improvements in robustness (tough gels), the proper dispersion/distribution of biopolymeric and synthetic polymers for proper interface bonding, conductivity improvements, and the additives’ impact on the electrolyte performance. There are several reasons for this. One is the obstacle to the whole field of biopolymers, i.e., the need for cost-effective and scalable biopolymer extraction technologies. The second one is that using biopolymers in combination with synthetic polymers is not a trivial task, even though the presence of functional groups (–OH, –NH_2_, –NHC(O)CH_3_, –COOH) in the biopolymer structures might allow for the design of targeted preferential interactions with both synthetic co-polymer and IL electrolyte, and even allow use of several biopolymers simultaneously or biopolymeric composites. Lastly, the device manufacturing approaches are still on the “traditional” side, although cost-effective, reproducible, and scalable methodologies such as 3D printing are available and might be adapted for the purpose.

Research efforts on the fabrication of biobased electronics are driven by increasing demand for wearable devices. The biocompatibility and biodegradability of biopolymers coupled with high voltage, which results in device miniaturization, will help realize supercapacitors for multiple applications, including powering implantable bioelectronics such as pacemakers and sensors. Such materials will combine high energy density, strength-to-weight ratio, and biocompatibility, and allow for scalable, rapid, and complex miniature fabrication. Still, a better understanding of the environmental and health impacts of all the components in the wearable EDLCs, including the IL itself, will be required before reaching commercialization.

## Figures and Tables

**Table 1:** Early representative examples of electrochemical properties of selected IL electrolytes.

Cation ^a^	Anion ^b^	T_m_, °C	T_dec_, °C	Density, g cm^−3^	Viscosity, cP	EW, V	Conduct., mS cm^−1^	Ref.
[Pyrr_1,3_]	[NTf_2_]	12	ND	1.45	61	5.3	3.9	[18,22]
	[N(SO_2_F)_2_]	−9	219	ND	40	5.3	8.2	[18,23]
	[N(SO_2_F)(SO_2_(C_2_F_5_))]	−99	343	1.44	56	6.0	3.5	[24]
[Pyrr_1,4_]	[NTf_2_]	−17.9	ND	1.41	85	6.0	2.2	[25,26]
	[NTf_2_]	ND	ND	1.39	83	5.5	2.8	[27]
[N_1113_]	[NTf_2_]	19	368	1.43	72	5.5	3.2	[27]
[Pip_1,4_]	[NTf_2_]	ND	371	1.38	155.3	2.2	1.1	[27]
[N_2222_]	[N(SO_2_F)(SO_2_(C_2_F_5_))]	6	324	1.44	104	6.0	1.5	[24]
[P_66614_]	[(C_2_F_5_)_3_PF_3_]	−50	ND	1.18	464	6.3	ND	[26,28,29]
	[NTf_2_]	−50	ND	1.18	464	5.2	ND	[30]

^a^ Cations. [Pyrr_1,3_]: *N*-methyl-*N*-propylpyrrolidinium; [Pyrr_1,4_]: *N*-methyl-*N*-butylpyrrolidinium; [N_1113_]: *N,N,N*-trimethyl-*N*-propylammonium; [Pip_1,4_]: *N*-methyl-*N*-butyl-piperidinium; [N_2222_]: Tetraethylammonium; [P_66614_]: Trihexyltetradecylphosphonium. ^b^ Anions. [N(SO_2_F)_2_]: bis(fluorosulfonyl)amide; [N(SO_2_F)(SO_2_(C_2_F_5_))]: (fluorosulfonyl)((perfluoroethyl)sulfonyl)amide; [NTf_2_]: bis(trifluoromethanesulfonyl)amide; [(C_2_F_5_)_3_PF_3_]: tri(pentafluoroethyl)trifluorophosphate.

**Table 2:** Biopolymer–IL electrolytes—Electrochemical properties ^a,b^.

Biopolymeric Matrix	Dopant IL	Ionic Conductivity (mS cm^−1^)	Supercapacitor Electrode	Capacitance	Ref.
PVA	[C_2_mim][EtSO_4_] + [NH_4_][OAc]	0.656	Reduced graphene oxide	138 F g^−1^	[65]
PVA/PVP	[N_4444_]I	Not reported	Laser-Induced Graphene	54.28 F g^−1^	[66]
Corn starch	Li[PF_6_] + [C_4_mim][PF_6_]	0.147 ± 0.02 (at 40 °C)	Activated carbon	36.79 F g^−1^	[62,67]
		0.199 ± 0.02 (at 80 °C)			[68]
	Li[PF_6_] + [C_4_mim][OTf]	0.600 ± 0.01 (at 80 °C)	Activated carbon	42.44 F g^−1^	[69]
		0.321 ± 0.01 (at 40 °C)			[62]
	NaCl + [C_6_mim]I	0.34 (16 wt% [C_6_mim]I)	Reduced graphene oxide	18.4 F g^−1^ (scan rate of 10 mV s^−1^); 24.8 F g^−1^ (low-frequency impedance)	[70]
	[NH_4_]I + [C_2_mim][SCN]	0.1 (12 wt% [C_2_mim][SCN])	Activated carbon	130 F g^−1^	[64]
Agarose functionalized (acetylated/carbanilated) in [C_4_mim][OAc]	[HEA][Formate] + [C_4_mim]Cl	0.848–1.200	Activated carbon	53 F g^−1^	[71]
Maltodextrin–MC–[NH_4_]Br	[C_2_mim]Br	0.339 ± 0.22 (at room temperature, 30 wt% IL)	Composite of carbon black, activated carbon, and PVdF	9.85 F g^−1^	[72]
Chitosan	acetic acid or adipic acid, [C_4_mim][BF_4_] + LiCl	2.91 (chitosan/adipic acid); 2.67 (chitosan/acetic acid)	Bucky paper	Not reported	[73]
MC	[Pyrr_1,4_][NTf_2_]	1.4 (at 30 °C), 6 (at 90 °C), 11.3 (at 140 °C)	Not tested	Not tested	[74]
MC	[C_2_mim][TCM]	19.3 (at 60 wt% IL)	Paste of porous carbon	38 F g^−1^ (at 5 mV S^−1^)	[75]
Cellulose or chitin/[C_2_mim][OAc]	[C_2_mim][BF_4_]	21.7 ± 3.5 (cellulose/[C_2_mim][BF_4_]); 22.2 ± 3.5 (chitin/[C_2_mim][BF_4_])	Activated carbon fiber cloth	140–145 F g^−1^ (capacitance retention ca. 90% after 10,000 galvanostatic charge-discharge cycles)	[63]
Chitosan/acetic acid/sodium hydroxide	[C_2_mim][BF_4_]	16.3 ± 0.2 (at 25 °C)	Activated carbon fiber cloths immersed in [C_2_mim][BF_4_]	131 F g^−1^ (stable for up to 5000 cycles)	[76]
Chitosan/hydroxyethyl methacrylate (HEMA)/[C_4_mim]Cl	[C_4_mim]Cl	31 to 81 (80 to 200 °C)	Activated charcoal	165 F g^−1^ (at 200 °C)	[77]
Chitin/[AMim]Br + Cellulose/[C_4_mim]Cl	[AMim]Br + [C_4_mim]Cl + H_2_SO_4_	578 (at 25 °C)	Activated carbon fiber cloths immersed in H_2_SO_4_	162 F g^−1^ (at 25 °C)	[78]
Sulfonated CNC/hyperbranched PILs	[C_2_mim][NTf_2_]	7.8 (95 wt% IL, at 30 °C)	Not tested	Not tested	[79]
CNFs/CNCs	[C_2_mim][NTf_2_]	4.3–6.2 (88–90 wt% IL)	Reduced graphene oxide films	34.5−44.1 F g^−1^ at 100 mV s^−1^	[80]
CNC-grafted-PMMA	[C_6_mim][NTf_2_]	5.7 ± 2.1 × 10^−3^ (at 30 °C)	Not tested	Not tested	[81]

^a^ Formulas: Cations: [C_2_mim]^+^: 1-ethyl-3-methylimidazolium; [C_4_mim]^+^: 1-butyl-3-methylimidazolium; [C_6_mim]^+^: 1-hexyl-3-methylimidazolium; Li^+^: lithium; Na^+^: sodium; [NH_4_]^+^: ammonium; [N_4444_]^+^: tetrabutylammonium; [HEA]^+^: *N*-(2-hydroxyethyl)ammonium; [Pyrr_1,4_]^+^: *N*-butyl-*N*-methylpyrrolidinium. Anions: [OAc]^−^: acetate; [NTf_2_]^−^: bis(trifluoromethylsulfonyl)imide; Br^-^: bromide; Cl^−^: chloride; [EtSO_4_]^-^: ethyl sulphate; [Formate]^−^: formate; I^-^: iodide; [PF_6_]^−^: hexafluorophosphate; [BF_4_]^−^: tetrafluoroborate; [SCN]^−^: thiocyanate; [TCM]^−^: tricyanomethanide. ^b^ Abbreviations: CNC: cellulose nanocrystals; CNF: cellulose nanofibers; MC: methylcellulose; PILs: polymeric ILs; PVP: polyvinylpyrrolidone; PVA: polyvinyl alcohol; PVdF: polyvinylidene fluoride.

## Data Availability

Data is contained within the article.

## References

[1] Liu C., Yu Z., Neff D., Zhamu A., Jang B.Z. (2010). Graphene-based supercapacitor with an ultrahigh energy density. Nano Lett..

[2] Beidaghi M., Wang C. (2012). Micro-supercapacitors based on interdigital electrodes of reduced graphene oxide and carbon nanotube composites with ultrahigh power handling performance. Adv. Funct. Mater..

[3] Pech D., Brunet M., Durou H., Huang P., Mochalin V., Gogotsi Y., Taberna P.-L., Simon P. (2010). Ultrahigh-power micrometre-sized supercapacitors based on onion-like carbon. Nat. Nanotechnol..

[4] Kim H.-K., Cho S.-H., Ok Y.-W., Seong T.-Y., Yoon Y.S. (2003). All solid-state rechargeable thin-film microsupercapacitor fabricated with tungsten co-sputtered ruthenium oxide electrodes. J. Vac. Sci. Technol. B.

[5] Wang K., Zou W., Quan B., Yu A., Wu H., Jiang P., Wei Z. (2011). An all-solid-state flexible micro-supercapacitor on a chip. Adv. Energy Mater..

[6] Beidaghi M., Wang C. (2011). Micro-supercapacitors Based on three dimensional interdigital polypyrrole/C-MEMS electrodes. Electrochim. Acta.

[7] Khomenko V., Frackowiak E., Béguin F. (2005). Determination of the specific capacitance of conducting polymer/nanotubes composite electrodes using different cell configurations. Electrochim. Acta.

[8] Brandt A., Pohlmann S., Varzi A., Balducci A., Passerini S. (2013). Ionic Liquids in Supercapacitors. MRS Bull..

[9] Wilkes J.S., Levisky J.A., Wilson R.A., Hussey C.L. (1982). Dialkylimidazolium chloroaluminate melts: A new class of room-temperature ionic liquids for electrochemistry, spectroscopy and synthesis. Inorg. Chem..

[10] Osteryoung R.A., Osteryoung J.G. (1987). Studies in ambient temperature ionic liquids. AFOSR Report 1987.

[11] Zhang L., Gao H., Jin G., Liu S., Wu J., Wu H., Yang Y., Wang Q., Wang S. (2022). Cellulose-Based Electrolytes for Advanced Lithium-Ion Batteries: Recent Advances and Future Perspectives. ChemNanoMat.

[12] Karuppasamy K., Theerthagiri J., Vikraman D., Yim C.J., Hussain S., Sharma R., Maiyalagan T., Qin J., Kim H.S. (2020). Ionic liquid-based electrolytes for energy storage devices: A brief review on their limits and applications. Polymers.

[13] MacFarlane D.R., Forsyth M., Howlett P.C., Kar M., Passerini S., Pringle J.M., Ohno H., Watanabe M., Yan F., Zheng W. (2016). Ionic liquids and their solid-state analogues as materials for energy generation and storage. Nat. Rev. Mater..

[14] Varela J.C., Sankar K., Hino A., Lin X., Chang W.-s., Coker D., Grinstaff M. (2018). Piperidinium ionic liquids as electrolyte solvents for sustained high temperature supercapacitor operation. Chem. Commun..

[15] Pitawala J., Navarra M.A., Scrosati B., Jacobsson P., Matic A. (2014). Structure and properties of Li-ion conducting polymer gel electrolytes based on ionic liquids of the pyrrolidinium cation and the bis (trifluoromethanesulfonyl) imide anion. J. Power Sources.

[16] Srour H., Rouault H., Santini C. (2012). Imidazolium based ionic liquid electrolytes for Li-ion secondary batteries based on graphite and LiFePO4. J. Electrochem. Soc..

[17] Mousavi M.P.S., Wilson B.E., Kashefolgheta S., Anderson E.L., He S., Bühlmann P., Stein A. (2016). Ionic liquids as electrolytes for electrochemical double-layer capacitors: Structures that optimize specific energy. ACS Appl. Mater. Interfaces.

[18] Galiński M., Lewandowski A., Stępniak I. (2006). Ionic liquids as electrolytes. Electrochim. Acta.

[19] Nancarrow P., Al-Othman A., Mital D.K., Dopking S. (2021). Comprehensive analysis and correlation of ionic liquid conductivity data for energy applications. Energy.

[20] Weingärtner H. (2014). The static dielectric permittivity of ionic liquids. J. Molec. Liquid.

[21] Kalb R.S., Shiflett M. (2020). Chapter 11: Toward Industrialization of Ionic Liquids. Commercial Applications of Ionic Liquids.

[22] Matsumoto H., Sakaebe H., Tatsumi K., Kikuta M., Ishiko E., Kono M. (2006). Fast cycling of Li/LiCoO_2_ cell with low-viscosity ionic liquids based on bis(fluorosulfonyl)imide [FSI]^−^. J. Power Sources.

[23] Zhou Q., Henderson W.A., Appetecchi G.B., Montanino M., Passerini S. (2008). Physical and electrochemical properties of n-alkyl-n-methylpyrrolidinium bis(fluorosulfonyl)imide ionic liquids: PY13FSI and PY14FSI. J. Phys. Chem. B.

[24] Liu K., Zhou Y.-X., Han H.-B., Zhou S.-S., Feng W.-F., Nie J., Li H., Huang X.-J., Armand M., Zhou Z.-B. (2010). Ionic liquids based on (fluorosulfonyl)(pentafluoroethanesulfonyl)imide with various oniums. Electrochim. Acta.

[25] MacFarlane D.R., Meakin P., Sun J., Amini N., Forsyth M. (1999). Pyrrolidinium imides:  A new family of molten salts and conductive plastic crystal phases. J. Phys. Chem. B.

[26] Ignat’ev N.V., Welz-Biermann U., Kucheryna A., Bissky G., Willner H. (2005). New ionic liquids with tris(perfluoroalkyl)trifluorophosphate (FAP) anions. J. Fluor. Chem..

[27] Abdallah T., Lemordant D., Claude-Montigny B. (2012). Are room temperature ionic liquids able to improve the safety of supercapacitors organic electrolytes without degrading the performances?. J. Power Sources.

[28] Yao C., Pitner W.R., Anderson J.L. (2009). Ionic liquids containing the tris(pentafluoroethyl)trifluorophosphate anion: A new class of highly selective and ultra hydrophobic solvents for the extraction of polycyclic aromatic hydrocarbons using single drop microextraction. Anal. Chem..

[29] Wibowo R., Aldous L., Rogers E.I., Ward Jones S.E., Compton R.G. (2010). A Study of the Na/Na+ redox couple in some room temperature ionic liquids. J. Phys. Chem. C.

[30] O’Mahony A.M., Silvester D.S., Aldous L., Hardacre C., Compton R.G. (2008). Effect of water on the electrochemical window and potential limits of room-temperature ionic liquids. J. Chem. Eng. Data.

[31] Zhou T., Zhao Y., Choi J.W., Coskun A. (2021). Ionic liquid functionalized gel polymer electrolytes for stable lithium metal batteries. Angew. Chem. Int. Ed..

[32] Dzulkipli M.Z., Karim J., Ahmad A., Dzulkurnain N.A., Su’ait M.S., Yoshizawa-Fujita M., Tian-Khoon L., Hassan N.H. (2021). The influences of 1-butyl-3-methylimidazolium tetrafluoroborate on electrochemical, thermal and structural studies as ionic liquid gel polymer electrolyte. Polymers.

[33] Pal P., Ghosh A. (2018). Solid-state gel polymer electrolytes based on ionic liquids containing imidazolium cations and tetrafluoroborate anions for electrochemical double layer capacitors: Influence of cations size and viscosity of ionic liquids. J. Power Sources.

[34] Lee D., Song Y.H., Choi U.H., Kim J. (2019). Highly flexible and stable solid-state supercapacitors based on a homogeneous thin ion gel polymer electrolyte using a poly(dimethylsiloxane) stamp. ACS Appl. Mater. Interfaces.

[35] Danyliv O., Strach M., Nechyporchuk O., Nypelö T., Martinelli A. (2021). Self-standing, robust membranes made of cellulose nanocrystals (CNCs) and a protic ionic liquid: Toward sustainable electrolytes for fuel cells. ACS Appl. Energy Mater..

[36] Ma D., Yuan D., Ponce de Leon C., Jiang Z., Xia X., Pan J. (2023). Current progress and future perspectives of electrolytes for rechargeable aluminum-ion batteries. Energy Environ. Mater..

[37] Hopson C., Villar-Chavero M.M., Dominguez J.C., Alonso M.V., Oliet M., Rodriguez F. (2021). Cellulose ionogels, a perspective of the last decade: A review. Carbohydr. Polym..

[38] Jamil R., Silvester D.S. (2022). Ionic liquid gel polymer electrolytes for flexible supercapacitors: Challenges and prospects. Curr. Opin. Electrochem..

[39] Sun L., Zhuo K., Chen Y., Du Q., Zhang S., Wang J. (2022). Ionic liquid-based redox active electrolytes for supercapacitors. Adv. Funct. Mater..

[40] Chen L., Fu J., Lu Q., Shi L., Li M., Dong L., Xu Y., Jia R. (2019). Cross-linked polymeric ionic liquids ion gel electrolytes by in situ radical polymerization. Chem. Eng. J..

[41] Wang X., Zhu H., Girard G.M.A., Yunis R., MacFarlane D.R., Mecerreyes D., Bhattacharyya A.J., Howlett P.C., Forsyth M. (2017). Preparation and characterization of gel polymer electrolytes using poly(ionic liquids) and high lithium salt concentration ionic liquids. J. Mater. Chem. A.

[42] Niu H., Ding M., Zhang N., Li X., Su X., Han X., Zhang N., Guan P., Hu X. (2023). Preparation of imidazolium based polymerized ionic liquids gel polymer electrolytes for high-performance lithium batteries. Mater. Chem. Phys..

[43] Sen S., Goodwin S.E., Barbará P.V., Rance G.A., Wales D., Cameron J.M., Sans V., Mamlouk M., Scott K., Walsh D.A. (2021). Gel–polymer electrolytes based on poly(ionic liquid)/ionic liquid networks. ACS Appl. Polym. Mater..

[44] Balo L., Gupta H., Singh S.K., Singh V.K., Tripathi A.K., Srivastava N., Tiwari R.K., Mishra R., Meghnani D., Singh R.K. (2019). Development of gel polymer electrolyte based on LiTFSI and EMIMFSI for application in rechargeable lithium metal battery with GO-LFP and NCA cathodes. J. Solid State Electrochem..

[45] Mathela S., Kumar S., Singh P.K., Chandra S.R., Shukla P., Singh V., Noor I., Kakroo S., Madkhli A.Y., Tomar R. (2022). Ionic liquid dispersed highly conducting polymer electrolyte for supercapacitor application: Current scenario and prospects—“ICSEM 2021”. High Perform. Polym..

[46] Osada I., de Vries H., Scrosati B., Passerini S. (2016). Ionic-liquid-based polymer electrolytes for battery applications. Angew. Chem. Int. Ed..

[47] Tamilarasan P., Ramaprabhu S. (2014). Stretchable supercapacitors based on highly stretchable ionic liquid incorporated polymer electrolyte. Mater. Chem. Phys..

[48] Liew C.W., Ramesh S., Arof A.K. (2014). Good prospect of ionic liquid based-poly(vinyl alcohol) polymer electrolytes for supercapacitors with excellent electrical, electrochemical and thermal properties. Int. J. Hydrogen Energy.

[49] Shi M.J., Kou S.Z., Shen B.S., Lang J.W., Yang Z., Yan X.B. (2014). Improving the performance of all-solid-state supercapacitors by modifying ionic liquid gel electrolytes with graphene nanosheets prepared by arc-discharge. Chin. Chem. Lett..

[50] Ujjain S.K., Sahu V., Sharma R.K., Singh G. (2015). High performance, all solid state, flexible supercapacitor based on Ionic liquid functionalized Graphene. Electrochim. Acta.

[51] Liew C., Ramesh S., Arof A.K. (2014). Characterization of ionic liquid added poly(vinyl alcohol)-based proton conducting polymer electrolytes and electrochemical studies on the supercapacitors. Int. J. Hydrogen Energy.

[52] Yang X., Zhang F., Zhang L., Zhang T., Huang Y., Chen Y.A. (2013). High-performance graphene oxide-doped ion gel as gel polymer electrolyte for all-solid-state supercapacitor applications. Adv. Funct. Mater..

[53] Pandey G.P., Hashmi S.A., Kumar Y. (2010). Performance studies of activated charcoal based electrical double layer capacitors with ionic liquid gel polymer electrolytes. Energy Fuels.

[54] Yun Y.S., Kim J.H., Lee S.Y., Shim E.G., Kim D.W. (2011). Cycling performance and thermal stability of lithium polymer cells assembled with ionic liquid-containing gel polymer electrolytes. J. Power Sources.

[55] Chinnam P.R., Zhang H., Wunder S.L. (2015). Blends of Pegylated Polyoctahedralsilsesquioxanes (POSS-PEG) and Methyl Cellulose as Solid Polymer Electrolytes for Lithium Batteries. Electrochim. Acta.

[56] Xiao S.Y., Yang Y.Q., Li M.X., Wang F.X., Chang Z., Wu Y.P., Liu X. (2014). A composite membrane based on a biocompatible cellulose as a host of gel polymer electrolyte for lithium ion batteries. J. Power Sources.

[57] Sudhakar Y.N., Selvakumar M. (2012). Lithium perchlorate doped plasticized chitosan and starch blend as biodegradable polymer electrolyte for supercapacitors. Electrochim. Acta.

[58] Xu D., Jin J., Chen C., Wen Z. (2018). From nature to energy storage: A novel sustainable 3D cross-linked chitosan–PEGGE-based gel polymer electrolyte with excellent lithium-ion transport properties for lithium batteries. ACS Appl. Mater. Interfaces.

[59] Song A., Huang Y., Zhong X., Cao H., Liu B., Lin Y., Wang M., Li X. (2017). Gel polymer electrolyte with high performances based on pure natural polymer matrix of potato starch composite lignocellulose. Electrochim. Acta.

[60] Kasprzak D., Galiński M. (2021). Chitin and chitin-cellulose composite hydrogels prepared by ionic liquid-based process as the novel electrolytes for electrochemical capacitors. J. Solid State Electrochem..

[61] Mittal N., Ojanguren A., Cavasin N., Lizundia E., Niederberger M. (2021). Transient rechargeable battery with a high lithium transport number cellulosic separator. Adv. Funct. Mat..

[62] Liew C.-W., Ramesh S. (2014). Comparing triflate and hexafluorophosphate anions of ionic liquids in polymer electrolytes for supercapacitor applications. Materials.

[63] Kasprzak D., Galiński M. (2022). Biopolymer-based gel electrolytes with an ionic liquid for high-voltage electrochemical capacitors. Electrochem. Commun..

[64] Konwar S., Singh A., Singh P.K., Singh R.C., Rawat S., Dhapola P.S., Agarwal D., Yahya M.Z.A. (2023). Highly conducting corn starch doped ionic liquid solid polymer electrolyte for energy storage devices. High Perform. Polym..

[65] Adarsh Rag S., Selvakumar M., Bhat S., Chidangil S., De S. (2020). Synthesis and characterization of reduced graphene oxide for supercapacitor application with a biodegradable electrolyte. J. Electron. Mater..

[66] Adarsh Rag S., Selvakumar M., De S., Chidangil S., Bhat S. (2022). Laser induced graphene with biopolymer electrolyte for supercapacitor applications. Mater. Today Proc..

[67] Ramesh S., Liew C.-W., Arof A.K. (2011). Ion conducting corn starch biopolymer electrolytes doped with ionic liquid 1-butyl-3-methylimidazolium hexafluorophosphate. J. Non-Cryst. Sol..

[68] Liew C.-W., Ramesh S. (2015). Electrical, structural, thermal and electrochemical properties of corn starch-based biopolymer electrolytes. Carbohydr. Polym..

[69] Liew C.-W., Ramesh S., Ramesh K., Arof A.K. (2012). Preparation and characterization of lithium ion conducting ionic liquid-based biodegradable corn starch polymer electrolytes. J. Solid State Electrochem..

[70] Ahuja H., Dhapola P.S., Rahul Sahoo N.G., Singh V., Singh P.K. (2020). Ionic liquid (1-hexyl-3-methylimidazolium iodide)-incorporated biopolymer electrolyte for efficient supercapacitor. High Perform. Polym..

[71] Trivedi T.J., Bhattacharjya D., Yu J.-S., Kumar A. (2015). Functionalized agarose self-healing ionogels suitable for supercapacitors. ChemSusChem.

[72] Asnawi A.S.F.M., Hamsan M.H., Aziz S.B., Kadir M.F.Z., Matmin J., Yusof Y.M. (2021). Impregnation of [Emim]Br ionic liquid as plasticizer in biopolymer electrolytes for EDLC application. Electrochim. Acta.

[73] Chupp J., Shellikeri A., Palui G., Chatterjee J. (2015). Chitosan-based gel film electrolytes containing ionic liquid and lithium salt for energy storage applications. J. Appl. Polym. Sci..

[74] Singh D., Kumar S., Singh A., Sharma T., Singh Dhapola P., Konwar S., Arkhipova E.A., Savilov S.V., Singh P.K. (2022). Ionic liquid–biopolymer electrolyte for electrochemical devices. Ionics.

[75] Yamagata M., Soeda K., Ikebe S., Yamazaki S., Ishikawa M. (2013). Chitosan-based gel electrolyte containing an ionic liquid for high-performance nonaqueous supercapacitors. Electrochim. Acta.

[76] Mantravadi R., Chinnam P.R., Dikin D.A., Wunder S.L. (2016). High conductivity, high strength solid electrolytes formed by in situ encapsulation of ionic liquids in nanofibrillar methyl cellulose networks. ACS Appl. Mater. Interfaces.

[77] Liu X., Wen Z., Wu D., Wang H., Yanga J., Wang Q. (2014). Tough BMIMCl-based ionogels exhibiting excellent and adjustable performance in high-temperature supercapacitors. J. Mater. Chem. A.

[78] Yamazaki S., Takegawa A., Kaneko Y., Kadokawa J.-I., Yamagata M., Ishikawa M. (2009). An acidic cellulose–chitin hybrid gel as novel electrolyte for an electric double layer capacitor. Electrochem. Commun..

[79] Lee H., Erwin A., Buxton M.L., Kim M., Stryutsky A.V., Shevchenko V.V., Sokolov A.P., Tsukruk V.V. (2021). Shape persistent, highly conductive ionogels from ionic liquids reinforced with cellulose nanocrystal network. Adv. Funct. Mat..

[80] Flouda P., Bukharina D., Pierce K.J., Stryutsky A.V., Shevchenko V.V., Tsukruk V.V. (2022). Flexible sustained ionogels with ionic hyperbranched polymers for enhanced ion-conduction and energy storage. ACS Appl. Mater. Interfaces.

[81] Kato R., Lettow J.H., Patel S.N., Rowan S.J. (2020). Ion-conducting thermoresponsive films based on polymer-grafted cellulose nanocrystals. ACS Appl. Mater. Interfaces.

[82] Yamazaki S., Takegawa A., Kaneko Y., Kadokawa J.i., Yamagata M., Ishikawa M. (2010). High/low temperature operation of electric double layer capacitor utilizing acidic cellulose–chitin hybrid gel electrolyte. J. Power Sources.

[83] Zheng S., Zhang J., Deng H., Du Y., Shi X. (2021). Chitin derived nitrogen-doped porous carbons with ultrahigh specific surface area and tailored hierarchical porosity for high performance supercapacitors. J. Bioresour. Bioprod..

[84] Dong D. (2019). Ternary Composite MnO_2_@MoS_2_/Polypyrrole from In-situ Synthesis for Binder-free and Flexible Supercapacitor. J. Bioresour. Bioprod..

[85] Wei L., Deng W., Li S., Wu Z., Cai J., Luo J. (2022). Sandwich-like chitosan porous carbon Spheres/MXene composite with high specific capacitance and rate performance for supercapacitors. J. Bioresour. Bioprod..

[86] Abdah M.A.A.M., Zubair N.A., Azman N.H.N., Sulaiman Y. (2017). Fabrication of PEDOT coated PVA-GO nanofiber for supercapacitor. Mater. Chem. Phys..

[87] Dong J., Wang Z., Kang X. (2016). The synthesis of graphene/PVDF composite binder and its application in high performance MnO_2_ supercapacitors. Colloids Surf. A Physicochem. Eng. Asp..

[88] Andres B., Dahlström C., Blomquist N., Norgren M., Olin H. (2018). Cellulose binders for electric double-layer capacitor electrodes: The influence of cellulose quality on electrical properties. Mater. Des..

[89] Rogers R.D., Zavgorodnya O., Shamshina J.L., Gurau G. (2018). Graphene-Biopolymer Composite Materials and Methods of Making Thereof. U.S. Patent.

[90] Choudhury N.A., Northrop P.W., Crothers A.C., Jain S., Subramanian V.R. (2012). Chitosan hydrogel-based electrode binder and electrolyte membrane for EDLCs: Experimental studies and model validation. J. Appl. Electrochem..

[91] Zhao Z., Cannon F.S., Nieto-Delgado C., Pena L. (2016). Lignin/collagen hybrid biomaterials as binder substitute for specialty graphites and electrodes. Carbon.

[92] Ghatee M.H., Moosavi F. (2011). Physisorption of hydrophobic and hydrophilic 1-alkyl-3-methylimidazolium ionic liquids on the graphenes. J. Phys. Chem. C.

[93] Murashko K., Nevstrueva D., Pihlajamäki A., Koiranen T., Pyrhönen J. (2017). Cellulose and activated carbon based flexible electrical double-layer capacitor electrode: Preparation and characterization. Energy.

[94] Ye W., Li X., Zhu H., Wang X., Wang S., Wang H., Sun R. (2016). Green fabrication of cellulose/graphene composite in ionic liquid and its electrochemical and photothermal properties. Chem. Eng. J..

[95] King C., Easton M.E., Rogers R.D. (2018). Chitin for the replacement of fluoropolymers in the assembly of electrochemical devices. ChemRxiv Camb. Camb. Open Engag..

[96] Liu Y., Wang Y., Nie Y., Wang C., Ji X., Zhou L., Pan F., Zhang S. (2019). Preparation of MWCNTs-graphene-cellulose fiber with ionic liquids. ACS Sustain. Chem. Eng..

[97] Lorenzo M., Srinivasan G. (2018). Durable flexible supercapacitors utilizing the multifunctional role of ionic liquids. Energy Technol..

[98] Li J., Wang N., Tian J., Qian W., Chu W. (2018). Cross-coupled macro-mesoporous carbon network toward record high energy-power density supercapacitor at 4 V. Adv. Funct. Mater..

[99] Nath G., Singh P.K., Dhapola P.S., Dohare S., Noor I.M., Sharma T., Singh A. (2022). Fabrication of cornstarch biopolymer-derived nano porous carbon as electrode material for supercapacitor application. Biomass Convers. Biorefinery.

[100] Suhas, Carrott P.J.M., Ribeiro Carrott M.M.L. (2007). Lignin-from natural adsorbent to activated carbon: A review. Bioresour. Technol..

[101] Babeł K., Jurewicz K. (2008). KOH activated lignin based nanostructured carbon exhibiting high hydrogen electrosorption. Carbon.

[102] Kijima M., Hirukawa T., Hanawa F., Hata T. (2011). Thermal conversion of alkaline lignin and its structured derivatives to porous carbonized materials. Bioresour. Technol..

[103] Klose M., Reinhold R., Logsch F., Wolke F., Linnemann J., Stoeck U., Oswald S., Uhlemann M., Balach J., Markowski J. (2017). Softwood lignin as a sustainable feedstock for porous carbons as active material for supercapacitors using an ionic liquid electrolyte. ACS Sustain. Chem. Eng..

[104] Krishnadoss V., Kanjilal B., Hesketh A., Miller C., Mugweru A., Akbard M., Khademhosseini A., Leijten J., Noshadi I. (2021). In situ 3D printing of implantable energy storage devices. Chem. Eng. J..

[105] Fang A., Smolyanitsky A. (2019). Large variations in the composition of ionic liquid-solvent mixtures in nanoscale confinement. ACS Appl. Mater. Interfaces.

[106] Kislenko S.A., Samoylov I.S., Amirov R.H. (2009). Molecular dynamics simulation of the electrochemical interface between a graphite surface and the ionic liquid [BMIM][PF6]. Phys. Chem. Chem. Phys..

[107] Kondrat S., Wu P., Qiao R., Kornyshev A.A. (2014). Accelerating charging dynamics in subnanometre pores. Nat. Mater.

[108] de Souza J.P., Pivnic K., Bazant M.Z., Urbakh M., Kornyshev A.A. (2022). Structural forces in ionic liquids: The role of ionic size asymmetry. J. Phys. Chem. B.

